# TGFβ signaling is associated with changes in inflammatory gene expression and perineuronal net degradation around inhibitory neurons following various neurological insults

**DOI:** 10.1038/s41598-017-07394-3

**Published:** 2017-08-09

**Authors:** Soo Young Kim, Vladimir V. Senatorov, Christapher S. Morrissey, Kristina Lippmann, Oscar Vazquez, Dan Z. Milikovsky, Feng Gu, Isabel Parada, David A. Prince, Albert J. Becker, Uwe Heinemann, Alon Friedman, Daniela Kaufer

**Affiliations:** 1 Department of Integrative Biology, University of California Berkeley, Berkeley, CA 94720 USA; 2 Helen Wills Neuroscience Institute, University of California Berkeley, Berkeley, CA 94720 USA; 3 Institute of Neurophysiology, Charité Universitätsmedizin Berlin, Berlin, D10117 Germany; 4 Departments of Cognitive and Brain Sciences, Physiology and Cell Biology, Zlotowski Center for Neuroscience, Ben-Gurion University of the Negev, Beer-Sheva, 84105 Israel; 5 Department of Neurology and Neurological Sciences, , Stanford University School of Medicine, Stanford, CA 94305 USA; 6 Department of Neuropathology, University of Bonn Medical Center, Bonn, 53105 Germany; 7 Department of Medical Neuroscience, Dalhousie University, Halifax, Nova Scotia B3H 4R2 Canada; 8 Canadian Institute for Advanced Research (CIFAR) Program in Child and Brain Development, ON M5G 1Z8 Toronto, Canada; 9 Carl-Ludwig-Institute for Physiology, Leipzig University, Leipzig, 04315 Germany

## Abstract

Brain damage due to stroke or traumatic brain injury (TBI), both leading causes of serious long-term disability, often leads to the development of epilepsy. Patients who develop post-injury epilepsy tend to have poor functional outcomes. Emerging evidence highlights a potential role for blood-brain barrier (BBB) dysfunction in the development of post-injury epilepsy. However, common mechanisms underlying the pathological hyperexcitability are largely unknown. Here, we show that comparative transcriptome analyses predict remodeling of extracellular matrix (ECM) as a common response to different types of injuries. ECM-related transcriptional changes were induced by the serum protein albumin via TGFβ signaling in primary astrocytes. In accordance with transcriptional responses, we found persistent degradation of protective ECM structures called perineuronal nets (PNNs) around fast-spiking inhibitory interneurons, in a rat model of TBI as well as in brains of human epileptic patients. Exposure of a naïve brain to albumin was sufficient to induce the transcriptional and translational upregulation of molecules related to ECM remodeling and the persistent breakdown of PNNs around fast-spiking inhibitory interneurons, which was contingent on TGFβ signaling activation. Our findings provide insights on how albumin extravasation that occurs upon BBB dysfunction in various brain injuries can predispose neural circuitry to the development of chronic inhibition deficits.

## Introduction

Brain damage due to stroke or brain trauma often yields neuronal hypersynchrony, potentially resulting in epilepsy and ensuing cognitive morbidities^1, 2^. Since no effective therapy exists for the prevention of injury-induced epilepsy, it is critical to identify key steps underlying the delayed pathological hyperexcitability. Insults to the brain commonly involve lasting dysfunction of the blood-brain barrier (BBB) formed by tight junctions between endothelial cells and ensures brain homeostasis by limiting the entry of blood-derived factors into brain parenchyma^3^. We have previously shown that chemically-induced disruption of the BBB induces pathological hyperexcitability manifested by epileptiform activity and recurring seizures^4^. We have further shown that exposure of the typically-secluded brain environment to albumin, the most abundant blood protein, is sufficient to induce epileptiform activity and recurring seizures, and this depends on activation of TGFβ signaling^4–7^. In the brain, albumin is uptaken into astrocytes, binds and activates TGFβ receptors, and can elicit aberrant rewiring of neural circuits including abnormal synaptic plasticity^8^ and excessive excitatory synaptogenesis^6^. However, a great deal is still unknown about the mechanisms by which BBB disruption and the consequent TGFβ signaling activation are linked with the delayed inhibitory circuit dysfunction following brain damage.

Despite differences in etiologies, clinical sequelae, and affected brain regions, striking similarities exist in the chronic deficits of inhibitory signaling, as repeatedly demonstrated in rodent injury models (summarized in Supplementary Table S1). Thus, we performed comparative analyses using human hippocampal samples as well as five different rat models of neurological insults in search of shared mechanisms. These include partial cortical isolation (“undercut”) as a model of traumatic brain injury^9, 10^, photothrombosis as a model of ischemic stroke^11, 12^, chemically-induced focal BBB disruption^4, 5^, direct brain exposure to serum albumin^4^ or TGFβ1^7^, and chronic pharmaco-resistant temporal lobe epilepsy in human patients. We show that a common transcriptional signature across rodent models predicts TGFβ-regulated extracellular matrix (ECM) remodeling. Accordingly, specialized ECM structures called perineuronal nets (PNNs) around fast-spiking inhibitory interneurons were degraded in the undercut rodent cortex. Next, infusion of albumin into a naïve mouse brain was used to test if albumin exposure is sufficient to induce the PNN degradation. Indeed, albumin lead to PNN degradation, and that was attenuated by losartan, previously shown to block TGFβ-signaling^13^. Finally, hippocampi resected from pharmaco-resistant temporal lobe epilepsy patients were analyzed. Similar to the mice and rat brains, there was an increase in TGFβ activation in astrocytes, and degradation of PNNs around fast-spiking inhibitory interneurons in epilepsy patients compared to age-matched controls. Our findings suggest that albumin-activated TGFβ signaling alters the microenvironment around inhibitory interneurons, as a potential mechanism underlying delayed deficits in neural inhibition following different injuries to the brain.

## Results

### Early blood-brain barrier disruption is evident in the undercut cortex and peri-infarct hippocampus

**Figure 1:**
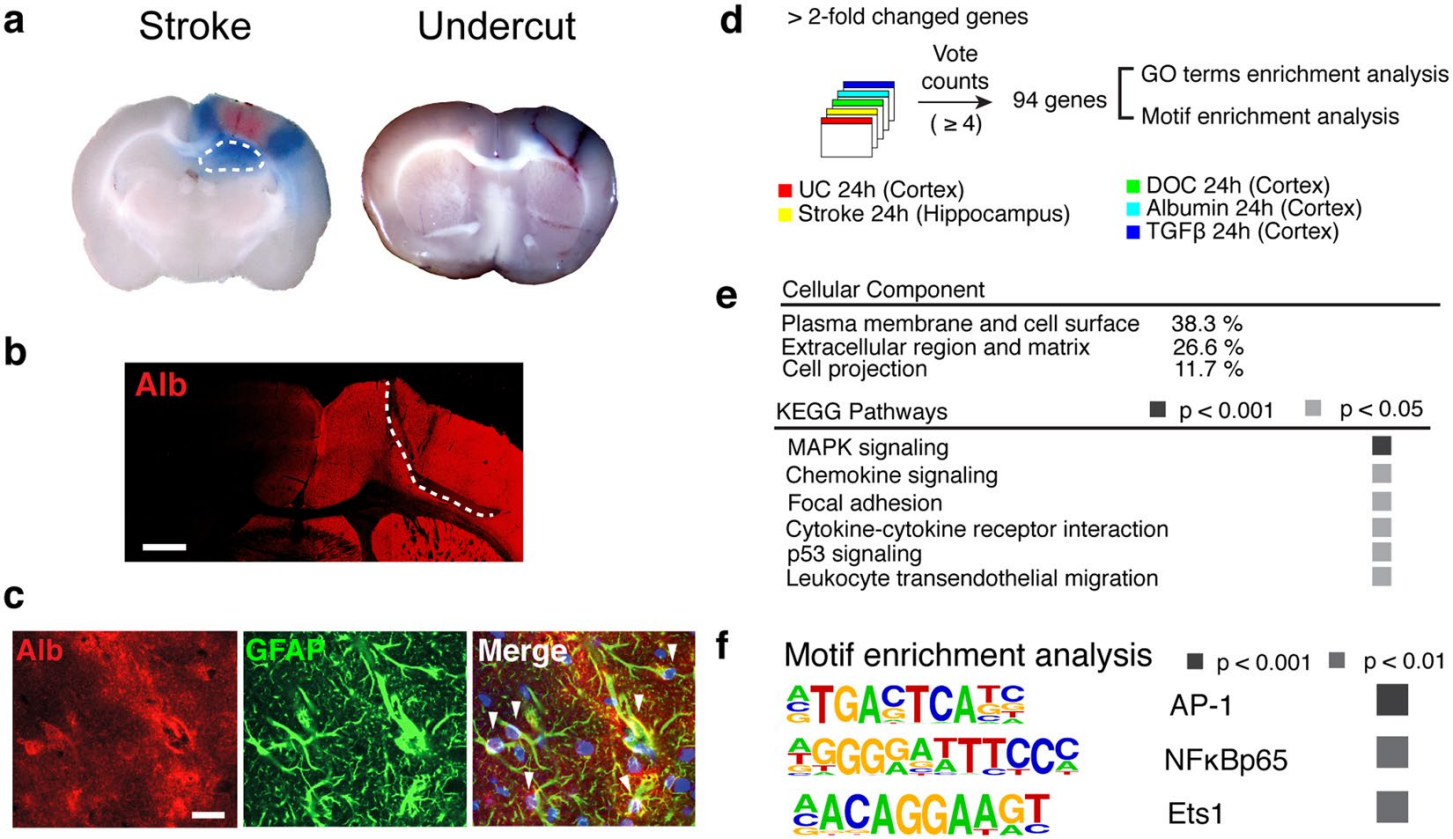
Comparative analysis revealed a common transcriptional signature in hyperexcitable regions following different insults. (**a**) Evans blue extravasation indicating BBB disruption in the peri-infarct hippocampus (outlined with white dotted line) and around the partially-isolated cortical regions 12 hours after stroke (left) and the undercut operation (right). (**b**) Representative micrograph of a rat brain slice immunostained for albumin (Alb) at 24 hours following the undercut (UC) operation (indicated by white dotted line). Scale bar = 1mm. (**c**) Representative confocal images of the UC cortex showing co-localization (white arrowheads) of albumin with GFAP (+) astrocytes. Nuclear staining with DAPI (blue) in the merged image. Scale bar = 20 μm. (**d**) A vote counting method for five independent microarray datasets from the undercut cortex (UC), peri-infarct hippocampus (Stroke), BBB-disrupted cortex treated with sodium deoxycholate (DOC), and cortices exposed to albumin (Alb) or TGFβ1 (TGFβ). (**e**) The gene ontology (GO) term enrichment analysis in the common transcriptional profile. (**f**) Using HOMER software, motifs including AP-1, NFκB, and ETS1 were found significantly enriched in the promoter proximal region, ±500 base pairs of the commonly upregulated genes across models.

BBB dysfunction had been previously reported to occur following TBI, stroke, and brain infections^2^. To test our hypothesis that BBB dysfunction is a precipitating event for injury-induced hyperexcitability, we first confirmed albumin extravasation in brain regions with hyperexcitable network activity in the injury models used in this study – peri-infarct hippocampus following photothrombotic cortical stroke^11, 12^ and partially isolated undercut cortex^9^. Evans-blue, an albumin-binding dye was intravenously administrated 12 hours after photothrombosis or the undercut surgery and detected in the peri-infarct cortex and hippocampus and around the partially-isolated cortical region (Fig. 1a). Immunostaining further confirmed albumin extravasation in the ipsilesional cortex at 24 hours following the undercut operations as well (Fig. 1b). Consistent with previous findings of albumin extravasation in rodent^6^ and human^14^ epileptic brains, albumin was co-localized within cells expressing glial fibrillary acidic protein (GFAP), a marker for astrocytes, in the undercut cortex (Fig. 1c).

### Functional annotation analysis reveals TGFβ-regulated transcriptional activation as an early common response to different insults

In search of commonality in the early transcriptional response to injuries and/or BBB dysfunction, we compared transcriptome profiles of the peri-infarct hippocampi and undercut cortices with three models for BBB dysfunction –BBB disruption induced by sodium deoxycholate (DOC) and direct cortical exposure to serum albumin or TGFβ1. Heat maps of genes annotated for inflammation and TGFβ signaling (Supplementary Fig. S1) show transcriptional induction. In order to identify a common transcriptional response across models, we used a vote counting method as previously described for large-scale meta-analysis^15^. Expression values for over 22,000 rat genes were scored and received a vote for each experiment in which the gene was dysregulated at 24 hours following insult (stroke, undercut, DOC, albumin, or TGFβ1). A total of 94 genes were found to be consistently upregulated across models (vote counts ≥ 4, Fig. 1d and Supplementary Fig. S2). Down regulated genes showed less consistency across models, with only two genes dysregulated in ≥4 conditions, and therefore were not further analyzed for functional annotation (Fig. S2). Annotation enrichment analysis on the list of upregulated genes identified plasma membrane, extracellular regions, and cell projections as enriched cellular components in the 94 genes (Fig. 1e, p’s < 0.05). The overrepresented Kyoto Encyclopedia of Genes and Genomes (KEGG) pathways included mitogen-activated protein kinase (MAPK) signaling, chemokine signaling, focal adhesion, cytokine-cytokine receptor signaling, indicating cell-matrix-cytokine interactions and massive inflammation as the common thread in early response to different injuries accompanying albumin extravasation (Fig. 1e).

We next explored which transcriptional factor motifs are enriched in the promoters of the commonly regulated genes. The motifs for AP1, NFκB (p65), and ETS1 were significantly overrepresented in the promoters of the commonly regulated genes (Fig. 1g). Both AP1, a Jun/Fos dimer transcription factor, and NFκB are known to be associated with Stat3 signaling^16^ as well as directly regulated by the TGFβ signaling pathway^17, 18^. ETS1 is an effector of the TGFβ signaling pathway^19^. Co-administration of TGFβ receptor blockers attenuated such transcriptional activation of majority of the commonly regulated genes upon albumin exposure (Fig. S3). These findings demonstrate that albumin-induced changes in gene expression are dependent on activation of TGFβ signaling pathways. To demonstrate the regulatory role of TGFβ in brain injury response, future experiments will need to directly test the effect of TGFβ blocker on ECM genes after brain injury.

### Transcriptional activation of extracellular matrix genes is evident across models

**Figure 2:**
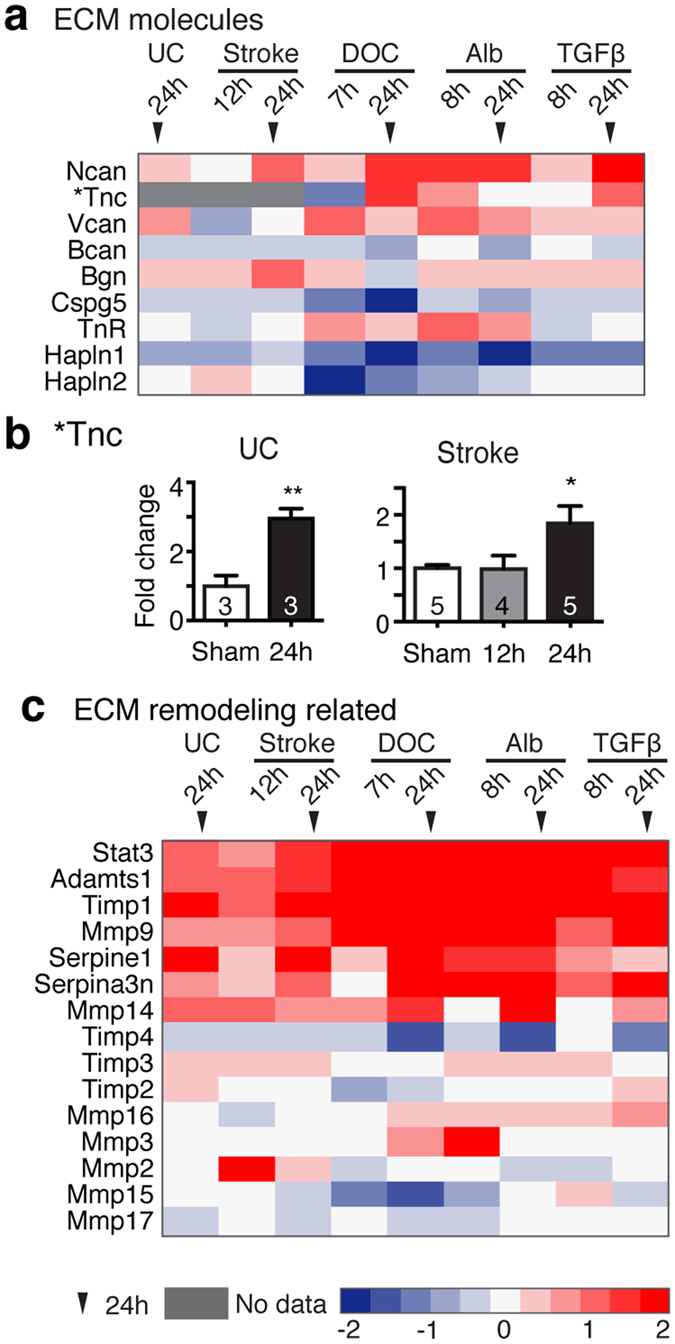
Transcriptional activation of TGFβ-regulated extracellular matrix genes across models. The expression levels of genes encoding for extracellular matrix (ECM) components (**a**) and for molecules involved in ECM remodeling (**c**) are shown across models. Heat maps are based on a log_2_ scale. (**b**) Quantitative real-time PCR was used to measure mRNA level of Tnc in the undercut (UC) cortex and peri-infarct hippocampi (Stroke). One way ANOVA with posthoc Turkey’s test (in Stroke, B) and student t-test (in UC). The number of animals used per condition is indicated within the bar.

One of the prominent events driven by the activation of TGFβ signaling is the modulation of extracellular matrix (ECM), which is essentially involved in epithelial to mesenchymal transition (EMT). EMT plays a central role in morphogenesis during early development and pathological processes such as tissue fibrosis and tumor invasion that require cell migration and ECM remodeling^18^. Likewise, AP1^17^, NF-κB^20^, ETS1^19^ and MAPK^17^ signaling pathways are key players in EMT-related ECM modulation. Given that ECM composition substantially affects synaptic function^21^, we hypothesized that ECM remodeling driven by TGF-β signaling following injuries might be associated with pathological hyperexcitability of neural networks occurring at a later time point. Therefore, we first explored the possibility that similar changes of ECM occur in the brain as a common response to epileptogenic injuries and BBB dysfunction. We examined the expression levels of selected genes that either encode ECM structural components or are involved in ECM remodeling across the five models (Fig. 2). The ECM components tenascin C (Tnc) and neurocan (Ncan) were highly increased 24 hours after all insults (Fig. 2a,b). In contrast, the expression of hyaluronan and proteoglycan link protein (Hapln) 1 that governs the structural integrity of ECM, was decreased at both 7/8 and 24 hours following chemically-induced BBB disruption and exposure to albumin or TGFβ1 (Fig. 2a). The expression of genes encoding for proteins involved in ECM degradation such as matrix metalloproteinase (MMP) 9, MMP14, and a disintegrin and metalloproteinase with thrombospondin type 1 (Adamts1), were consistently upregulated across models (Fig. 2c). Together, these transcriptional changes predict a considerable remodeling of the ECM.

### ECM surrounding fast-spiking inhibitory interneurons is degraded in the injured cortex

**Figure 3:**
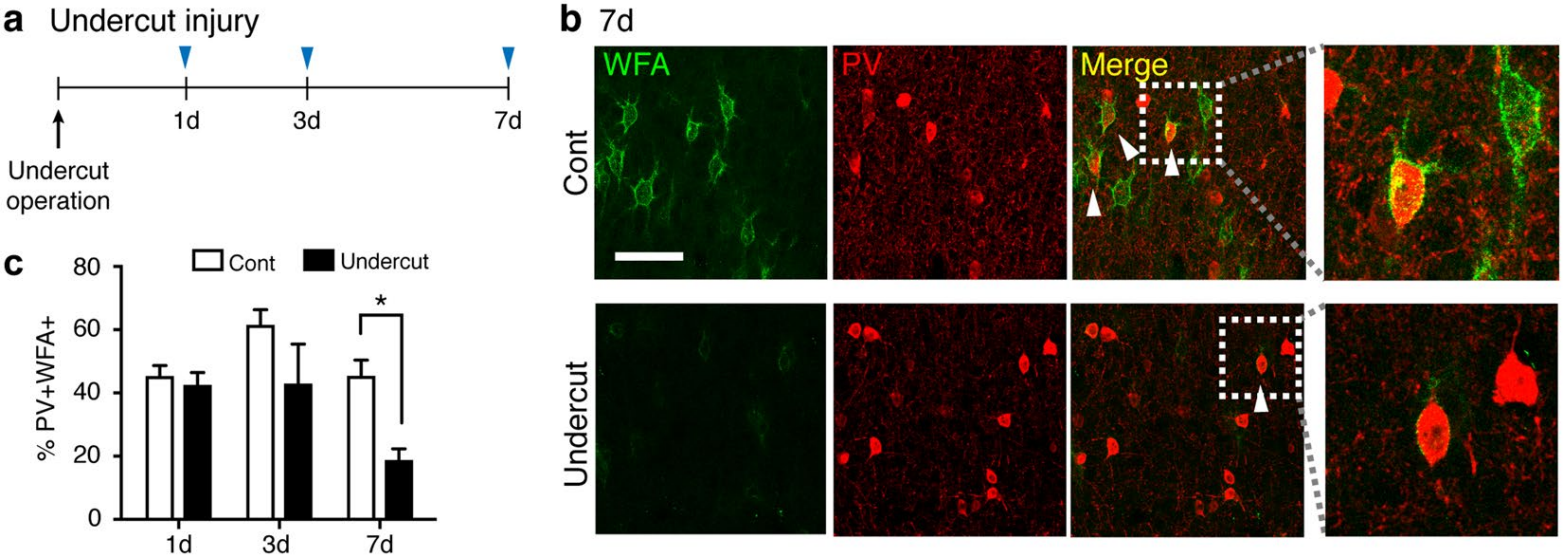
Perineuronal nets around PV(+) interneurons are degraded following traumatic brain injury. (**a**) Time line of experimental design. (**b**) Representative confocal images of rat undercut and contralateral (Cont) cortices stained for parvalbumin (PV) and perineuronal nets using *Wisteria Floribunda* agglutinin (WFA) 7 days after the operation. Scale bar = 50 µm. White arrowheads indicate representative PV(+)/WFA(+) cells. Areas within white rectangles in merged images are shown magnified. (**c**) The percentage of PV(+) interneurons associated with PNNs was significantly decreased at 7 days following the undercut operation. *p < 0.05, Two-way ANOVA with post-hoc Sidak’s test, two tailed. Three biological replicates (n’s = 3) were used at each time point.

This predicted ECM alteration is interesting as it may be associated or even causatively involved in chronic deficits of inhibitory signaling and pathological hyperexcitability observed following injury. Particularly, a specialized ECM structure named perineuronal nets (PNNs)^22^ is tightly associated with parvalbumin (PV) expressing fast-spiking inhibitory interneurons. These interneurons play a critical role in the regulation of hyperexcitable network activity, and are responsible for the generation of gamma oscillations, enabling coordinated activation of ensembles of principal cells^23^. Functional deficits of fast-spiking interneurons are a key pathological factor in epilepsy^24^ and are also found in the injured brain. Indeed, it has been previously reported that reduced perisomatic inhibition and structural abnormality of fast-spiking interneurons are seen in the undercut cortex^9, 10^. Gamma oscillations are reduced in the peri-infarct hippocampus following ischemic cortical stroke as well^12^. We first examined whether PNN degradation occurs in the injured brain. Co-staining of *wisteria floribunda agglutinin* (WFA), a broad marker labeling PNNs, with immunostaining for parvalbumin (PV), a marker for fast-spiking interneurons revealed significant main effects of time and undercut treatment on the percentage of PV(+)/WFA(+) interneurons (Fig. 3a,b and Supplementary Fig. S3). The percentage of PV(+)/WFA(+) cells out of a total number of PV(+) interneurons was significantly decreased in the undercut cortex at 7 days following injury, compared to the contralateral homotopic region (Fig. 3c). It should be noted that a total number of WFA(+) cells were decreased at 7 days following injuries (Contralateral vs. Ipsilateral, 451.44 ± 47.78 vs. 243.78 ± 37.23 cells per mm^2^, p < 0.05, two-tailed student t-test), while the percentage of non-PV cells labeled with WFA in the somatosensory cortex was not significantly altered by the undercut injuries (%PV(−)WFA(+) out of WFA (+) cells, Contralateral vs. Ipsilateral, 58.4 ± 6.98 49.5 ± 9.94, p = 0.491, two-tailed). This implies that not only PNNs around PV cells are degraded, but the ECM structures labeled with WFA around non-PV cells might be also affected by injury.

### Albumin increases the expression of ECM genes in astrocytes via TGFβ/Alk5 pathway

**Figure 4:**
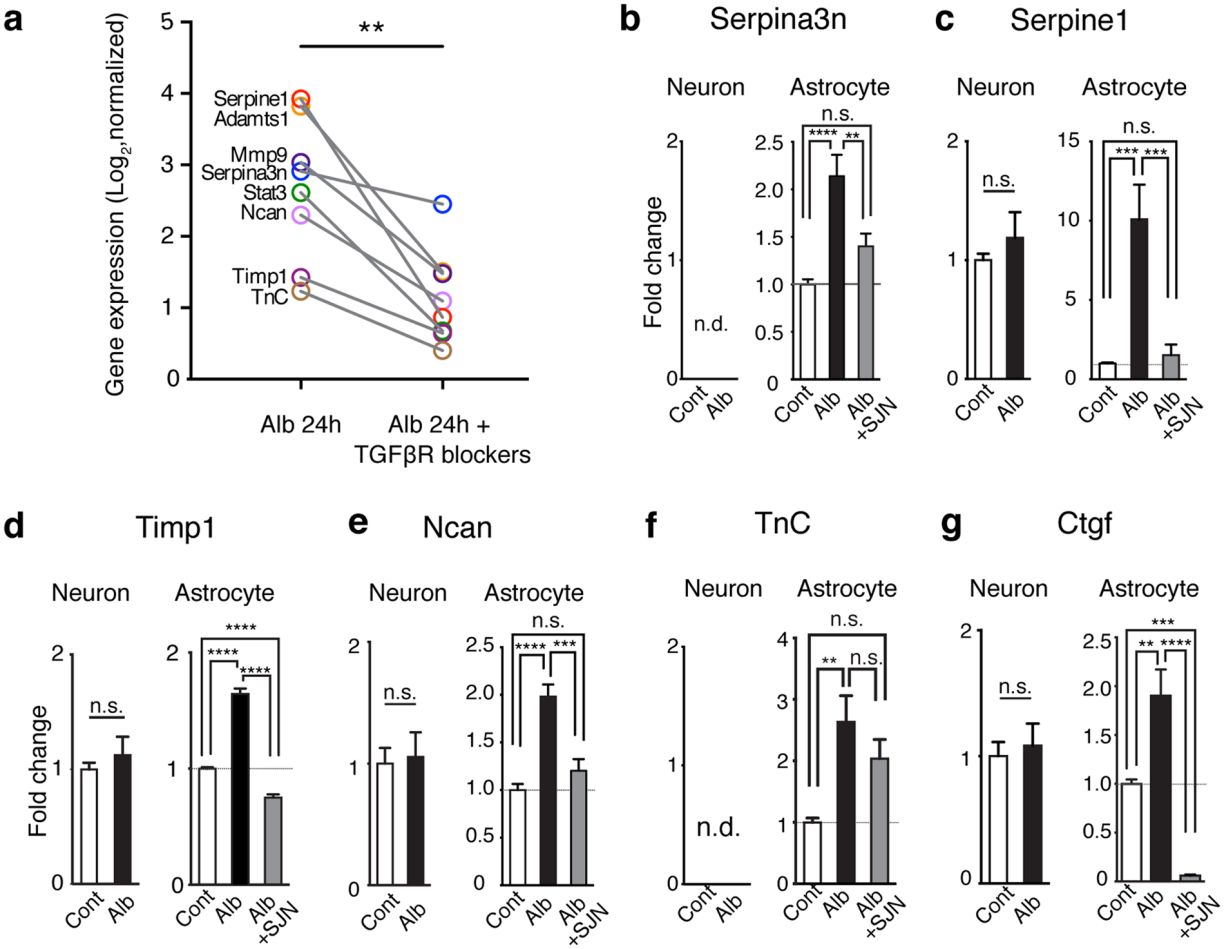
Albumin activated the transcriptional responses of ECM genes via TGFβ receptor signaling. (**a**) The expression levels of ECM-related genes is upregulated across models (marked in bold in A, C) in a separate microarray data set in comparisons between albumin vs. albumin + TGFβ receptor (R) blockers. **p = 0.0078, Wilcoxon signed-rank test, two-tailed. (**b**–**g**) Quantitative real-time PCR for the mRNA expression of selected genes in primary cortical astrocytic or neuronal cultures at 24 hours following treatment of albumin or albumin plus a specific Alk5 blocker, SJN2511. Results were of three independent primary culture derivations (n = 11 per condition in neurons; n = 10–12 per condition in astrocytes). One way ANOVA with posthoc Turkey’s test (in astrocytes) and student t-test (in neurons) were performed. Cont, control; Alb, albumin; SJN, SJN2511; n.d., non detectable (Cq value ≥ 35). *p < 0.05, **p < 0.01, ***p < 0.001, ****p < 0.0001. Data are shown as mean ± S.E.

Previously we have shown that albumin activates astrocytic TGFβ signaling via TGFβ receptor type I activin receptor-like kinase 5 (Alk5), leading to the increased phosphorylation of Smad2/3, the proximate effector of TGFβ signaling pathways in astrocytes, but not in neurons^4^. We therefore next examined whether the effect of albumin on those genes are mediated by TGFβ-receptor signaling, using cortical tissue collected 24 hrs following exposure to albumin or albumin plus TGFβ receptor blockers (as in ref. 7). The expression level of eight ECM genes robustly upregulated across the five models (shown in Fig. 2) was attenuated in the presence of TGFβ receptor blockers (Fig. 4d and Fig. S4), suggesting that albumin-induced activation of ECM genes requires the activation of TGFβ receptor-mediated signaling.

Next, we asked what is the cellular source of the ECM-related transcriptional response. We treated primary rat cortical cultures enriched for astrocytes or neurons with albumin for 24 hours. Following albumin exposure, the mRNA levels of Serpina3n, Serpine1, Timp1, Ncan, and Tnc, were significantly increased in astrocytes (Fig. 4b–f), consistent with the results in the *in vivo* models. In contrast, albumin exposure did not modulate their mRNA levels in neuronal cultures (Fig. 4b–e). Additionally, the mRNA expression of connective tissue growth factor (CTGF), a key regulator for TGFβ-mediated EMT by governing the transcription of ECM molecules^18^, was also increased in astrocytes but not in neurons upon albumin exposure (Fig. 4g). Albumin-induced transcriptional modulation in cultured astrocytes was blocked in the presence of the selective ALK5 blocker, SJN2511(Fig. 4b–g).

### Albumin exposure is sufficient to induce persistent degradation of ECM around fast-spiking inhibitory interneurons

**Figure 5:**
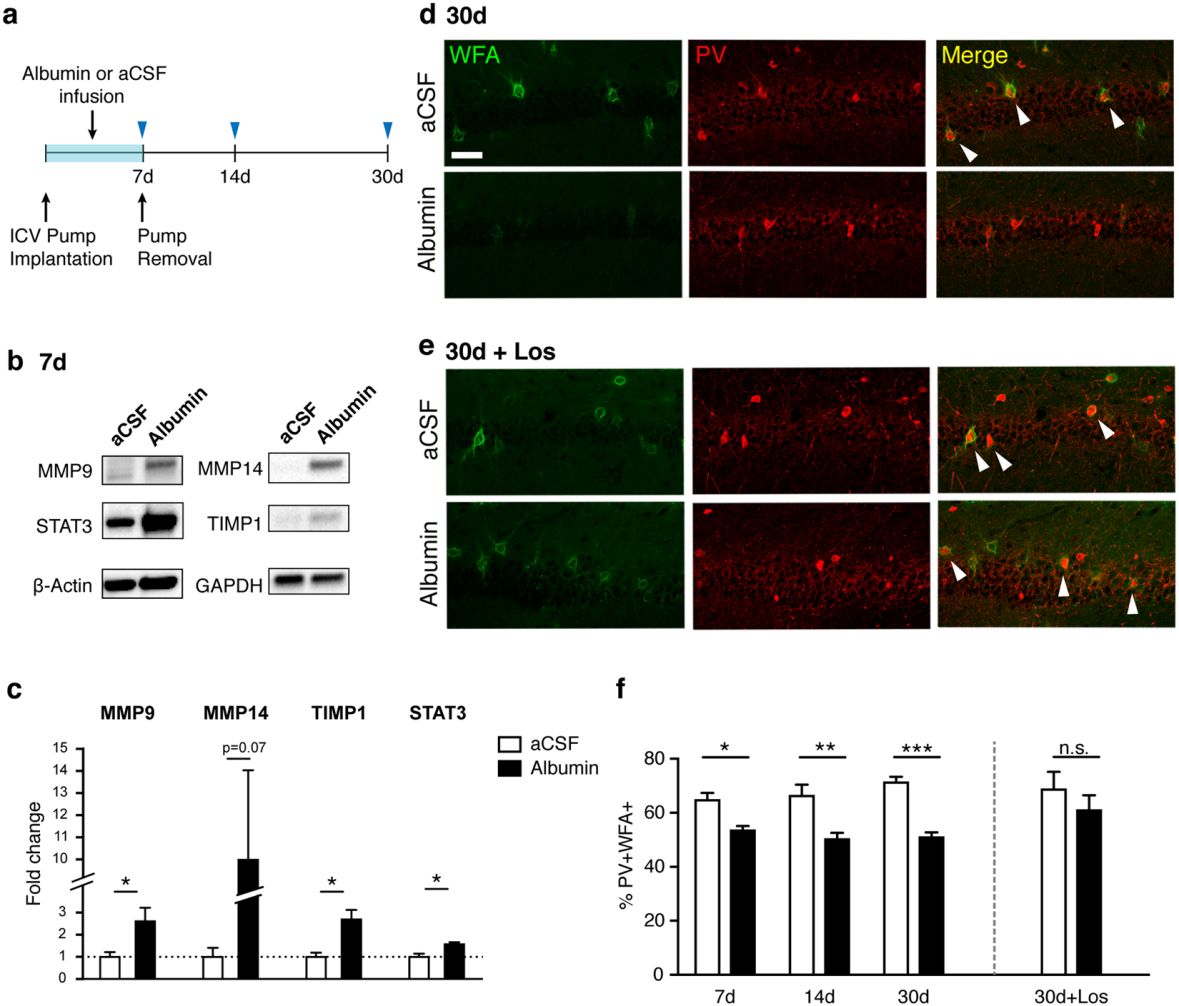
Albumin induced the degradation of perineuronal nets around PV(+) interneurons and losartan attenuated the effect of albumin. (**a**) Experimental design. Intracerebroventricular (*ICV*) osmotic pumps containing albumin (0.4 mM) or aCSF were implanted in rats and mice and removed after 7 days. (**b,c**) Molecules involved in ECM remodeling including MMP9, TIMP1, and STAT3 were significantly increased in dissected rat hippocampi following albumin infusion for 7 days. Uncropped blot images are available in the Supplementary Fig. S4. Each group has four biological replicates. Unpaired student t- test. *p < 0.05, two-tailed. (**d**) Representative confocal images showing PNNs (WFA) and PV(+) cells in the mouse hippocampal CA1 region at 30 days post implantation. (**e**) A separate set of animals was infused with albumin + losartan (10μM) or aCSF + losartan. Scale bar = 50 μm. (**f**) Albumin exposure decreased the association of PNNs with PV(+) interneurons (*p < 0.05, **p < 0.01, ***p < 0.001, Two-way ANOVA, post-hoc Sidak’s test, two tailed). Co-administration of losartan attenuated the effect of albumin (p > 0.05, Mann-Whitney test, two-tailed). n.s., not significant. Each condition has three or four biological replicates (n = 3–4).

We next addressed the question whether albumin extravasation was responsible for the PNN degradation observed in the injured brain. To test that, we exposed mice to albumin intracerebroventricular (*icv*) infusion. Animals were implanted with *icv* osmotic pumps delivering albumin or artificial cerebrospinal fluid (aCSF) for 7 days and sacrificed at different time points post implantation (Fig. 5a). This model was chosen as we have previously shown that it enables the chronic controlled infusion of albumin to the hippocampus and induces spontaneous seizures^6^. First, we investigated whether the chronic exposure to albumin induces changes in proteins involved in ECM remodeling, as predicted by the transcriptional analysis using the *in vivo* models (Fig. 2). Western blot analysis revealed significantly increased levels of MMP9, TIMP1, and STAT3 proteins in the hippocampus of animals that received albumin infusion compared with those exposed to aCSF (Fig. 5b–c). Accordingly, Mmp9, Timp1, and Stat3 mRNA levels were also upregulated across the different *in vivo* models (Fig. 2 and Supplementary Fig. S2). MMP14 protein level was not statistically significant different in albumin-infused vs. control animals.

Next, we tested whether albumin exposure by itself is sufficient to induce degradation of PNNs around fast-spiking interneurons. We estimated the percentage of PV(+) cells that are enwrapped by WFA(+) ECM structures in the hippocampus of mice at different time points following pump implantation. Consistent with findings in the rat undercut cortex, the percentage of PV(+)/WFA(+) interneurons in the mouse hippocampus was significantly decreased following 7 days of albumin exposure, compared to control mice infused with aCSF (Fig. 5b and Supplementary Fig. S5). Decreases in the percentage of PV(+)/WFA(+) cells in albumin-infused hippocampi were persistently found 23 days after the removal of osmotic pumps (Fig. 5b,c). These findings suggest that exposure of a naïve brain to albumin is sufficient to result in chronic PNN degradation, as found in the injured cortex.

### Losartan blocks albumin-induced chronic degradation of ECM around PV(+) interneurons

Losartan, an FDA approved angiotensin II type 1 receptor antagonist, has been shown to inhibit TGFβ signaling-driven ECM remodeling^13, 25^. We have previously shown that losartan blocks albumin-induced TGFβ signaling and prevents the development of recurring seizures^4^. We implanted mice with osmotic pumps that delivered *icv* infusion of losartan with albumin or aCSF for 7 days and examined the association between PNNs and PV(+) interneurons in the hippocampus at 30 days post implantation. In the presence of losartan, the percentage of PV(+)/WFA (+) interneurons in animals treated with albumin was not different from controls infused with aCSF (Fig. 5b and d), indicating that losartan administration blocked albumin-induced PNN degradation around PV+ cells.

### Activated astrocytic TGFβ signaling and PNN degradation are found in hippocampal sections from patients with chronic epilepsy

**Figure 6:**
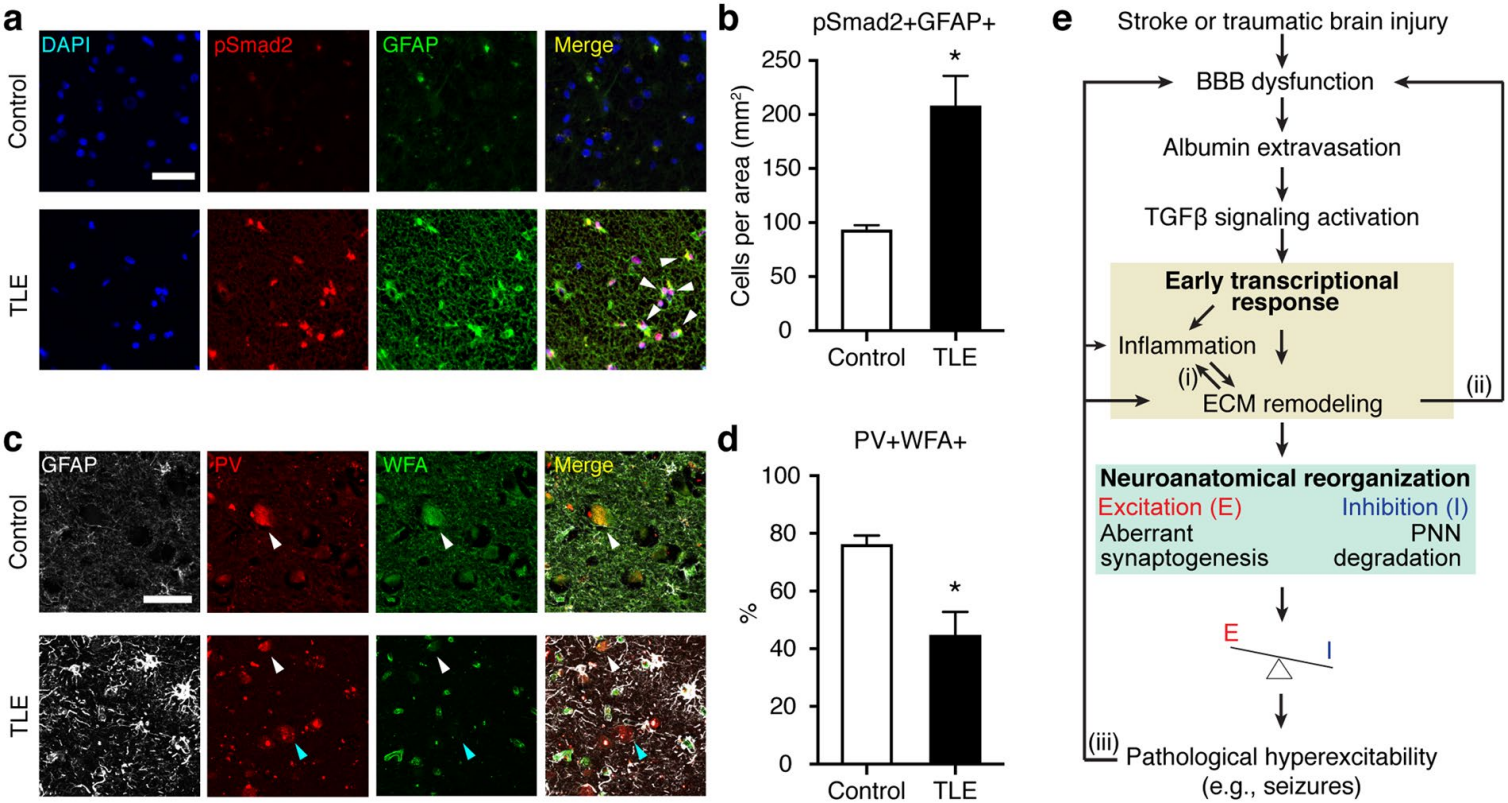
Increased expression of astrocytic pSmad2 and reduced association of PNNs around PV(+) cells in human epileptic hippocampi. (**a**) Representative micrographs of human hippocampi stained for GFAP and phosphorylated Smad2, a downstream effecter of TGFβ signaling. The hippocampi were resected from temporal lobe epilepsy (TLE) patients or age-matched autopsy controls. Representative GFAP(+) astrocytes expressing pSmad2(+) are indicated by white arrowheads. Scale bar = 50 μm. (**b**) Co-localization of pSmad2 with GFAP was increased in TLE patients (n = 5) compared to controls (n = 3). (**c**) Representative confocal micrographs of human hippocampal tissues stained for GFAP, PV, and PNNs (WFA) resected from TLE patients or controls. Scale bar = 50 μm. White and blue arrowheads indicate representative PV(+)/WFA(+) and PV(+)/WFA(−) cells, respectively. (**d**) The percentage of PV(+)/WFA(+) cells was decreased in the hippocampus of TLE patients (n = 4) compared to controls (n = 4). Mann-Whitney test, two-tailed, was used for statistical analyses. *p < 0.05. (**e**) A working model. Several points for feed-forward loops (i-iii) can lead to chronic hyperexcitability and the development of epilepsy. The dysfunction of blood-brain barrier (BBB) and the ensuing entry of albumin into brain parenchyma activate TGFβ signaling. Comparative transcriptome analyses predicted the activation of the common core signaling transduction including MAPK pathway, Stat3, NFκB, AP-1, and ETS1. These signaling pathways can elicit the reciprocal activation of inflammation and extracellular matrix (ECM) remodeling (i). ECM remodeling can trigger the transformation of a latent from of TGFβ to its active form^75^ as well as exacerbate BBB dysfunction^58^ (ii). The degradation of perineuronal nets (PNNs) around fast-spiking interneurons and aberrant excitatory synaptogenesis occur in the course of ECM remodeling, presumably leading to functional alterations in inhibition and abnormality in synaptic plasticity that may contribute to excitation/inhibition (E/I) imbalance and ultimately the occurrence of seizures. Finally, seizures *per se* cause BBB dysfunction, inflammation, and the upregulation of MMPs activity^31, 43^ (iii).

Because BBB dysfunction and albumin uptake into astrocytes were previously demonstrated in the hippocampi of temporal lobe epilepsy (TLE) patients^14^, we predicted that albumin-induced TGFβ signaling activation and subsequent PNN degradation may also occur in the hippocampus of TLE patients. Phosphorylation of Smad2 (pSmad2) proteins was quantified as a marker of TGFβ receptor-mediated signaling in hippocampal tissues resected from TLE patients (detailed information is available in Supplementary Table S2) and autopsy controls. The number of GFAP (+) astrocytes co-localized with pSmad2 protein was significantly increased in hippocampal sections of TLE patients compared to controls (Fig. 6a and b). Consistent with our findings in the rodent brains the percentage of PV(+)/WFA(+) interneurons was significantly decreased in the hippocampus resected from TLE patients in compared with controls (Fig. 6c and d). These results corroborate the potential role of TGFβ-regulated ECM remodeling in the pathology of chronic hyperexcitability.

## Discussion

The present study was designed to address the question whether different types of injuries (with different etiologies) involve acute transcriptional responses, and if so, do any common changes emerge that can explain inhibitory deficits known to commonly be found following neurological insults including stroke, traumatic brain injury, and BBB breakdown? We compared transcriptional responses at an early time point (within 24 h) following different types of insults. We identified a common transcriptional response including genes encoding for key molecules involved in ECM remodeling and found the induction of proteins of a subset of those genes as a result of albumin exposure *in vivo*. Indeed, PNN degradation was apparent at least 7 days after insults and persistent until 30 days post initial insult.

Given its association with a wide range of cellular processes, TGFβ signaling pathway has long been a therapeutic target in many diseases including various types of cancer. Here, we show that the common transcriptional activation predicts TGFβ-regulated ECM remodeling as a common response to different insults, and the degradation of perineuronal nets (PNNs), an ECM structure tightly associated with fast-spiking inhibitory interneurons is found in the injured brain at a later time point. Albumin extravasation into the brain parenchyma, which often occurs when BBB function is compromised, emerges as a potential regulator, as exposure to albumin alone was sufficient to induce similar transcriptional changes and degradation of PNNs around PV+ interneurons. Considering the critical role of ECM in synaptic plasticity and function^21, 26^ as well as the known effect of albumin on excitatory synaptogenesis^6^, we propose a working model for the development of chronic hyperexcitability following brain damage (Fig. 6e). BBB dysfunction, evident after various types of injuries, allows the entry of albumin into the brain. Albumin induces an early transcriptional response via activation of TGFβ receptors, specifically related to inflammation and ECM remodeling. This in turn potentially generates a microenvironment promoting neuroanatomical reorganization, including aberrant excitatory synaptogenesis and PNN degradation around inhibitory interneurons, that all together can potentially lead to the development of pathological hyperexcitability.

The signaling pathways that emerge as activated across models in the present study- MAPK, NFκB, AP-1, and ETS1 - are well known to play a significant role in inflammation^16, 27–29^. Recently, inflammation has gained much interest as a possible therapeutic target for the prevention of epilepsy development due to its role in the pathological progression^30, 31^. Inflammation accompanies massive migration of cells and cytokines, which not only requires, but also elicits ECM remodeling^32^. The tight association between ECM remodeling and inflammation had been reported in the central nervous system (CNS). For example, in the inflamed spinal cord, ECM components such as neurocan and tenascin C are abnormally upregulated, while hyaluronan and aggrecan, the main components maintaining scaffolding and matrix structure, are progressively degraded, concomitant with the persistent upregulation of matrix metalloproteinases (MMPs)^33^. Interestingly, neurocan and tenascin C are not only abundantly expressed by reactive astrocytes following brain injury^34^, but also greatly upregulated following status epilepticus^35, 36^. Therefore, our finding that albumin increases mRNA levels of tenascin C and neurocan in astrocytes via TGFβ/Alk5 pathway supports a critical role of astrocytic TGFβ signaling in inflammation-coupled changes of ECM during epileptogenesis.

Emerging evidence highlights the importance of ECM for synaptic plasticity^21, 37^. While in the present study we quantified PNNs around fast-spiking inhibitory interneurons, it should not be ruled out that albumin extravasation and subsequent transcriptional responses in ECM-related genes may affect ECM structures around different types of cells and alter synaptic plasticity of other types of neurons besides PV(+) interneurons. For instance, the upregulation of MMP9 has been found to induce excessive excitatory synaptogenesis in rodent epilepsy models^38^. Given our previous finding that albumin increases excitatory synaptogenesis via TGFβ signaling^6^, such ECM remodeling may contribute to excitatory synaptogenesis. It has been previously reported that hippocampal neurons derived from transgenic mice that lack four ECM molecules – the glycoproteins, Tenascin C and R, and the chondroitin sulfate proteoglycans (CSPGs), brevican and neurocan – shows abnormalities in synaptic structure and function^21^. Upregulation of astrocyte-derived tenascin C is associated with neurite outgrowth following injury^39^. Therefore, it seems reasonable to speculate that ECM remodeling would affect not only PV(+) cells but also various types of neurons and their synaptic plasticity. Notably, it has been reported that although PNNs labeled by WFA are found primarily around PV(+) interneurons, they are also found surrounding other types of cells including glutamatergic neurons^40^. Indeed, we have found WFA(+)/PV(−) cells both in the injured cortex (Fig. 3) and hippocampus chronically exposed to albumin (Fig. 5). Given the heterogeneity in PNNs and the cell types surrounded by them^41^, alterations in ECM could result in a broad spectrum of changes in synaptic function and neural connectivity. Further studies are necessary to dissect how ECM remodeling driven by albumin extravasation differentially affects various types of cells.

Previous studies reported the association between PNNs and hyperexcitable networks. The administration of kainic acid, an epileptogenic agent, was recently shown to induce changes in components of PNNs, which hampers the maturation of PV(+) cells in the rodent cortex and hippocampus^42^. PNNs around PV(+) interneurons in the rat hippocampus were found to be degraded following status epilepticus induced by pilocarpine, concomitant with the increased activity of MMPs^43^, and inhibition of MMPs can prevent seizure development as well as PNN degradation in kindled rats^44^. A recent study by Hsieh and colleagues reported that PNN degradation preceded PV(+) cell impairment and loss of cortical inhibition following traumatic brain injury^45^, corroborating our hypothesis that PNN degradation predisposes injured brains to inhibitory dysfunction. Given our finding that albumin exposure *per se* can drive the chronic degradation of PNNs, we suggest that BBB dysfunction occurring in the injured brain contributes to injury-induced PNN degradation and eventually deficits of inhibition.

The pathological consequences of degraded PNNs and the electrophysiological traits of PV(+) interneurons following the loss of PNNs are still largely unknown. Technical limitations hinder manipulating PNNs exclusively due to the overlap in the components of PNNs and other ECM structures^46^. However, the functional roles of PNNs during the early postnatal development have been extensively studied. For example, PV(+) interneurons play a critical role in the maturation of developing local circuits in the visual cortex^47^. PNNs specifically capture and transfer othodenticle homeobox 2 (Otx2), an essential protein for the maturation of PV(+) interneurons^48^ into the cells^49^. It has been reported that Otx2 has a neuroprotective effect by restoring mitochondrial dysfunction in mice retinas^50^. Furthermore, PNNs are found to protect PV(+) interneurons against oxidative stress in rodent prefrontal cortex^51^. In fact, PV(+) interneurons are particularly vulnerable to oxidative stress due to their intrinsic electrophysiological properties that feature high energy demand and require a high level of mitochondrial oxidative capacity^52^. A recent study showed that the increased level of oxidative stress was concomitantly found with a gradual loss of cortical inhibition following brain injury^45^. Notably, brain injuries as well as seizures induce heightened oxidative stress along with chronic inflammation^2, 30, 31^. Therefore, it is plausible to speculate that the chronic loss of PNNs due to BBB disruption renders the PV(+) interneurons more susceptible to mitochondrial dysfunction against oxidative stress, leading to functional deficits of those cells and reduced inhibition in local circuits. Further study is required to elucidate the pathological role of degraded PNNs around PV(+) interneurons and to examine whether the heightened level of oxidative stress is a key factor linking PNN degradation with chronic deficits of inhibitory signaling following injuries.

In addition to its preventive effect on epileptogenesis via TGFβ suppression^4^, losartan blocked the effect of albumin on the association of PNNs with PV(+) interneurons. As mentioned above, given that seizures can induce increased inflammation and MMP activity^43^, we cannot rule out the possibility that the effect of losartan on PNNs may in part result from its suppression of seizures. However, it has been repeatedly demonstrated that losartan normalizes pathological changes of ECM via TGFβ suppression^13, 25, 53^, supporting that its potent inhibitory effects on TGFβ-induced ECM remodeling may directly attenuate the albumin-induced degradation of PNNs.

Notably, there are conflicting reports in regards to TGFβ signaling, as being neuroprotective or neurotoxic. Prehn and colleagues (1993) reported that the administration of TGFβ1 prior to the onset of ischemic stroke reduces neuronal damage^54^. Transgenic mice whose astrocytic TGFβ signaling is inhibited were shown to have increased inflammation and reduced functional outcome following stroke compared to wild-type mice^55^. On the other hand, transgenic mice over-expressing TGFβ1 in astrocytes developed seizures and overexpression of ECM molecules^56^. Blocking TGFβ-Smad2/3 signaling in innate immune cells reduced deposits of cerebrovascular beta amyloid in the Alzheimer’s disease model^57^. While the increased circulating TGFβ1 has been found to exacerbate BBB permeability in a mouse model of hepatic encephalopathy via upregulation of MMP9^58^, it has been also reported that macrophages play a critical role in maintaining integrity of BBB following stroke via TGFβ1^59^. These conflicting findings seem to highlight the context-dependent role of post-injury TGFβ signaling. Given that the current findings on the linkage between TGFβ signaling and PNN degradation around inhibitory interneurons point strongly to a possible use of TGFβ blockers as a preventive therapy for acquired epilepsy, further investigation is required to elucidate the mechanisms underlying the beneficial and detrimental effects of TGFβ signaling in the pathological sequelae following brain damage.

In line with the context-dependent role in injury-induced pathology, some preclinical studies have reported the neuroprotective effect of albumin in rodent stroke models, suggesting albumin exposure as a potential clinical therapy^60, 61^. Indeed, there was a large-scale clinical trial to test its beneficial effects in stroke patients. According to a recent report^62^, however, albumin administration has no therapeutic benefits and is in fact associated with greater adverse events such as increased rates of intracerebral hemorrhage or pulmonary edema. Although such discrepancy between preclinical evidence and the clinical trial remains unexplained yet^62^, the effect of albumin exposure shown in the present study might contribute in part to its adverse effect. We used albumin at a concentration up to the one corresponding to 100% of serum concentration (0.4 mM) with an assumption of maximum disruption of BBB. Given that stroke involves BBB dysfunction and its extent may vary with infarct sizes, additional albumin administration for stroke patients may induce massive albumin exposure to broad brain regions, resulting in adverse events including intracerebral hemorrhage. Furthermore, a temporal window of albumin exposure may be a critical factor. In the preclinical study of albumin administration in rodent stroke models, the beneficial effect of albumin administration on post-stroke brain swelling was significant only when albumin was administered within 4 hours after the onset of ischemic stroke^60^. In contrast, post-stroke albumin extravasation manifested by Evans Blue injections seemed to be maximal at 12 hour after the onset of photothrombosis and gradually normalized^11^. Moreover, subsequent transcriptional responses to the injury was greater at 24 h compared to 12 h following the onset of stroke in the present study, suggesting that the effect of albumin on ECM remodeling might be delayed. Further studies are required to elucidate the pathological role of albumin with a high temporal resolution following injuries and its long-term consequences.

Upon dysfunction of the BBB, multiple blood-borne elements other than albumin gain access to the normally-secluded brain environment, and can potentially initiate a sequence of pathological events. For example, fibrinogen from the blood can trigger reactive astrocytosis and increase the expression of neurocan and other scar-forming molecules via the activation of TGFβ signaling^63^. Furthermore, iron has long been regarded as a potential trigger for chronic neurodegeneration, particularly found in multiple sclerosis^64^. It has been reported that chronic hemorrhage arising from cerebral cavernous malformation often present with epilepsy in patients^65^. These findings corroborate that the entrance of blood-borne molecules is associated with neuroinflammation and pathological hyperexcitability. Furthermore, as we proposed in our working hypothesis (Fig. 6e), a sequence of processes from TGF-β regulated transcriptional responses can elicit several feedforward loops to manifest delayed worsening of BBB integrity that possibly leads to chronic exposure of the brain parenchyma to blood-borne molecules. Since it has been demonstrated that exposure of the brain environment to albumin in the absence of injury or BBB dysfunction is sufficient to recapitulate inflammation, neurodegeneration, and epileptogenesis (summarized in Supplementary Table S1), we focused on the downstream signaling of albumin extravasation in the present study.

BBB dysfunction is tightly associated with other neurodegenerative disorders including Alzheimer’s disease and multiple sclerosis^66^, both of which involve chronic deficits of PV(+) interneurons^67, 68^. In particular, such deficits of inhibitory interneurons were found to be a critical factor for abnormal network activity and cognitive dysfunction in a rodent model of Alzheimer’s disease^68^. Furthermore, a gradual disruption of BBB has been recently demonstrated in the aging human hippocampi and this was tightly associated with mild cognitive impairment^69^, highlighting a potent role of the chronic BBB disruption in aging-related cognitive dysfunction. Therefore, our findings on ECM remodeling upon albumin extravasation may provide a clue on pathogenic mechanisms for a broad spectrum of neurological disorders that involve BBB dysfunction.

## Methods

### Animals

All experimental procedures were approved by the animal care and use ethical committees at the University of California Berkeley, Stanford University, Charité University Medicine Berlin, and Ben-Gurion University of the Negev, Beer Sheva and were performed in accordance with the appropriate guidelines and regulations. Partial isolation of sensorimotor cortex (“Undercut”) was made in 15 male Sprague-Dawley rats (1 month old). Animals were deeply anesthetized with ketamine and xylazine and the scalp of was incised. Cranial window over somatosensory motor cortex was made and a 30-gauge needle, bent 90° 2mm from the tip, was inserted parasagittally, advanced under direct vision tangentially, and lowered 2mm. The needle was then rotated 180° and elevated to beneath the pial vessels, and removed. A second transcortical cut was made parallel and 2mm laterally to the first. Animals with sham operations underwent craniectomy only without inserting a needle. For RNA extractions, the brain region underneath the cranial window was dissected 24 hour after the operation and fresh frozen (Sham, n = 3; Undercut, n = 3). For immunohistochemistry, animals were transcardially perfused at 24 hour, 3 days, and 7 days post operation (n’s = 3). For stroke surgeries, 15 male Wistar rats (240–350 g) were used and photothrombotic stroke onto somatosensory cortex was made. Animals were anesthetized with ketamine and xylazine (i.p., 1.6 and 0.6 mg/kg, respectively). The skin was opened and a halogen light beam (diameter 3.5 mm) was directed for 15 min onto the intact skull (1 mm posterior and lateral to bregma) after intravenous injection of Rose bengal (20 mg/kg). Sham operation (n = 5) consisted of either exposure to a light beam (n = 3) or injection of Rose Bengal (n = 2). Peri-infarct hippocampi from animals with stroke were dissected and fresh frozen at 12 hour (n = 4) and 24 hour (n = 5) post operations. Sham-operated animals were sacrificed 12 hour (n = 2, one with light beam and the other with Rose Bengal) and 24 hour (n = 3, one with light beam and two with Rose Bengal) after sham operations. To examine whether blood-brain barrier disruption occurs in hippocampus following photothrombotic stroke in rats, we intravenously administered Evans blue, which binds to intrinsic albumin, to animals immediately after the induction of photothrombosis and checked for extravasation after 12 hours, as previously described^11^.

### Intracerebroventicular osmotic pump implantation

Surgeries were performed on 29 male C57BL/6 J mice (3 months old, used for immunihistochemistry) or 14 wistar male rats (10 weeks old, used for Western blot analysis) under isofluorane anesthesia (1–2%). Using a sterotaxic frame, a 0.7 mm diameter hole was drilled through the skull over the somatosensory cortex (0.5 mm caudal, 1 mm lateral to bregma) and anterior to the hippocampus. The pumps (ALZET, Cupertino, CA) were filled with 200 μL of either bovine serum albumin (0.2 mM for rats as previously described^4^, 0.4 mM for mice, Sigma-Aldrich, St. Louis, MO) solution or 0.4 mM bovine serum albumin mixed with 10 µM losartan in artificial cerebrospinal fluid (aCSF) as previously described^5^, and placed subcutaneously between the shoulder blades. We used a concentration of albumin up to 0.4 mM in the *icv* administration corresponding to 100% of serum albumin concentration^5, 70^. Controls were implanted with pumps containing aCSF. Pumps delivered their contents into the right lateral cerebral ventricle via a brain infusion kit (ALZET).

### RNA extraction and microarray analysis

Total RNA was isolated using TRIzol reagent (Invitrogen, Grand Island, NY). The quality of RNA was checked by using the Agilent 2100 Bioanalyzer (Agilent Technologies, Santa Clara, CA). Samples were prepared and hybridized to Rat 2.0 ST Arrays (Affymetrix, Santa Clara, CA) according to manufacturer’s protocols. Data were normalized and processed as described previously^7^. Briefly, data were preprocessed using the log2 transformation and quantile normalization with the RMA algorithm. Differentially expressed genese were defined as showing a greater than 2 fold change compared to the sham-operated groups in each condition, while a minimum expression value of 5 in at least one of the conditions being compared. Gene expression data are available on the Gene Expression Omnibus (GEO, http://www.ncbi.nlm.nih.gov/geo; accession number GSE81302). The microarray data sets from the previous study^7^ were downloaded from GEO (accession number GSE12304). The previous study used three different types of BBB dysfunction models: the use of BBB disrupting agent, deoxycholic acid (DOC, 2 mM), direct application of albumin (0.1 mM, corresponding to 25% of serum albumin cocentration) or TGFβ1 (at a concentration of 10 ng /mL). Those concentrations were determined based on the effectiveness to induce hyperexcitability^7^. The web-based program, Database for Annotation, Visualization, and Integrated Discovery (DAVID, http://david.abcc.ncifcrf.gov/) was used for functional annotation analysis^71^. Genes showing consistent changes across all experimental conditions were identified using a vote counting method as previously described in meta-analysis^15^. While down-regulated genes were less consistent across models with only two genes (Abcg2 and Egr1, shown in Fig. S2) downregulated in 4 or more conditions, upregulated genes were more consistent yielding a list of 94 dysregulated in 4 or more conditions (Fig. S2). Functional annotation analyses were performed on this set of 94 genes including gene ontology (GO) enrichment and motif discovery. Expression levels of a subset of those genes were verified using quantitative RT-PCR (Fig. S1). Redundant GO terms of cellular components are combined (e.g., extracellular region and extracellular region part). The promoter proximal motif search was performed using HOMER v 4.7, a software program for ChIP-Seq analysis (http://homer.salk.edu/homer/)^72^. The promoter proximal region was defined as ±500 base pairs of the promoter of interest. Heat maps were made using MATLAB.

### Primary rat cortical astrocyte and neuron cultures

For primary rat neuronal cultures, cerebral cortices were collected from embryonic day 18 rats and cells were dissociated using papain solution (10 U/mL, Sigma-Aldrich). Mechanical trituration was performed to attain single cell suspension that was then plated. Four hours after incubation, the culture medium was replaced with Neurobasal medium (Life Technologies) supplemented with 2% B27 and 0.5 mM GlutaMax (Life Technologies). For primary rat astrocytic cultures, cells from cerebral cortices of 2-day-old rat pups were prepared as previously reported. Cells were dissociated, plated in flasks, and cultured in high-glucose DMEM supplemented with 10% fetal bovine serum and 1% penicillin/streptomycin. After 10 days, flasks were shaken to remove microglia and cells were passaged onto plates. Astrocyte cultures were serum-deprived for 18 hour prior to albumin treatment by replacing the medium with serum-free high-glucose DMEM supplemented with 1% penicillin/streptomycin. Bovine serum albumin (BSA with a minimum of 98%, fraction V; Sigma-Aldrich) in serum-free media was then given for 24 hours with the final concentration of 0.2 mM. SJN2511 (30 µM, final concentration; obtained from Tocris Biosciences, Minneapolis, MN), a TGFβ receptor I blocker, was added 1 hour prior to BSA treatment. All cells were maintained in 5% CO_2_ at 37 °C.

### Real time quantitative PCR

The expression level of mRNA was detected by real time quantitative reverse transcriptase-PCR using CFX96 Real Time System (BioRad) with SsoAdvanced SYBR Green Supermix (BioRad). Primer sequences are available in Supplementary Information.

### Western blot analysis

Tissue was homogenized and protein lysates were extracted from rat hippocampal samples using RIPA buffer (50 mM Tris-HCl, 150 mM NaCl, 1% NP-40, 0.5% Sodium deoxycholate, 0.1% SDS) including a protease (Calbiochem #539134) and phosphatase inhibitor cocktail (Roche PhoStop Ref: 4906845001). Protein samples were run under reducing conditions–20 μg of protein lysate was mixed with Laemmli buffer (Bio-Rad #161–0737), containing 5% 2-mercaptoethanol (Sigma M6250), and fractionated by SDS-PAGE using the Mini-PROTEAN® Tetra System and pre-cast TGX™ Gels (Bio-Rad #456-1096); Spectra™ Multicolor Broad Range Protein Ladder Thermo #26634) was used as a reference. Following separation, samples were transferred to a nitrocellulose membrane (0.45 µm, Bio-Rad #1620115). Membranes were blocked for 1 hr at room temperature with 5% non-fat dry milk (Apex #20-241) in TBST (10 mM Tris,150 mM NaCl, 0.5% Tween 20, pH 8.0), and incubated overnight at 4 °C with primary antibody. The next day, membranes were washed with TBST and incubated with appropriate secondary antibodies for 1 hour at room temperature. Membranes were washed with TBST and visualized using chemiluminescence SuperSignal West Dura Extended Substrate (Thermo #34075) and Bio-Rad Chemidoc system with Bio-Rad Image Lab software (version 4.0.1). Restore PLUS Stripping beffer (Thermo Fischer #46430) was used to strip blots of in between antibody sets, before probing with new ones. The amount of proteins was quantified with densitometry analysis using Image J. Detailed information of antibodies used for Western Blot analysis are available in Supplementary Information.

### Immunohistochemistry and quantification

Following transcardial perfusion with 4% paraform aldehyde, coronal brain sections (40 µm) of rats were cut with a sliding microtome (Microm HM400, Heidelberg). For mice brain sections (20 µm), brains were frozen and cut with CryoStar NX70 cryostat (ThermoFisher Scientific). Tissues were pretreated with 0.3% Triton-X in PBS for 30 min, followed by 1-hour incubation with normal donkey serum, and then incubated overnight with primary antibodies at 4 °C, followed by a series of washing and the incubation with secondary antibodies at room temperature.

Chicken polyclonal anti-albumin (Abcam, AB106582; 1:300) and mouse monoclonal anti-parvalbumin (Sigma P3099, 1:1000) were used. Flourescein-labeled *Wisteria Floribunda* agglutinin (WFA, VectorLabs, 1:400) was used to detect perineuronal nets (PNNs). For secondary antibodies, we used cy3-anti-Chicken (Jackson ImmunoResearch Laboratories; 1:500) and Cy3-anti mouse (Jackon ImmunoResearch Laboratories; 1:500). Images were taken Zeiss 710 confocal microscope with 2 µm-optical slices. For quantification, three sections (200 µm apart) per animal were included for quantification. Region of interest (ROI) was traced at low magnification using Stereoinvestigator (MBF Biosciences, Williston, VT). For rat brain sections, boundaries of partially isolated undercut cortices as well as contralateral homotopic somatosensory cortices were identified as ROI. For mice brain sections, cornu ammonis (CA) 1–3 areas were traced at low magnification since the *icv* albumin is infused into these areas of hippocampus as previously shown^6^. The percentage of PV(+)/WFA(+) cells among overall PV(+) cells was used as a measure of PNNs’ association with PV(+) cells. Experimenters were blinded to conditions throughout.

### Human tissue

All participants gave informed and written consent and all procedures were conducted in accordance with the Declaration of Helsinki and approved by the local ethics committees of the individual institutions. For histopathological examination, resected hippocampi of patients and autopsy control tissue (Supplementary Table S2) were cut into 5 mm thick coronal slices along the anterior–posterior axis.

Tissue samples were fixed in 4% formalin and routinely processed into liquid paraffin. All specimens were cut at 4 µm with a microtome (Microm, Heidelberg, Germany), stretched in water at 40 °C and mounted on slides coated with silane (Langenbrinck). The slides were subsequently air-dried in an incubator at 37 °C overnight, deparaffined in descending alcohol concentration and stained with hematoxylin and eosin (HE) and HE-luxol-fast blue. Diagnostic procedures comprised various further routinely used immunohistochemical reactions including antibodies against glial fibrillary acidic protein (GFAP), synaptophysin, neurofilament protein (NFP) as described previously^73, 74^. After heat-induced antigen retrieval with sodium citrate buffer (pH 6.0), we used following antibodies: mouse monoclonal anti-parvalbumin (Sigma, P3088; 1:200), chicken polyclonal anti-glial fibrillary acidic protein (Millipore, AB5541; 1:400), mouse monoclonal anti-GFAP (Cell Signaling, #3670; 1:50), rabbit polyclonal anti-pSmad2 (Millipore, AB3849; 1:2000), Cy3 anti-mouse IgG, Alexa647, anti-chicken IgG, Alexa488 anti-mouse IgG, and Cy3 anti-rabbit IgG. All secondary antibodies were obtained from Jackson ImmunoResearch Labs and used at the dilution of 1:200. For quantification, images from 10 of randomly selected sampling areas per patient were collected using Zeiss Axio Observer Z1 epifluorescence microscopy equipped with Metamorph software for automatic cell scoring and image capturing. Experimenters were blinded to conditions throughout.

### Statistical analysis

Prism6 (GraphPad) was used for statistical analysis. For mRNA data, one-way analysis of variance (ANOVA) was used with Turkey’s post-hoc test. For immunohistochemistry, two-way ANOVA was used with Sidak’s post-hoc test if significant main (time or condition) or interaction effects were detected. Wilcoxon signed-rank test was used for the differential expression of eight ECM genes in matched-pair comparisons between array data sets. Mann-Whitney test was used to examine the effect of losartan in mice brains and used for human tissue analysis without assumption of normal distribution. Effects were considered significant at p < 0.05. Results are presented as the mean ± SE.

Additional detailed information on experimental procedures is available in Supplementary Information.

## Electronic supplementary material

Supplementary info
