# Supplementary material for: TGFβ signaling is associated with changes in inflammatory gene expression and perineuronal net degradation around inhibitory neurons following various neurological insults
Source: Sci Rep. 2017 Aug 9;7:7711. doi: 10.1038/s41598-017-07394-3 (PMC5550510; doi:10.1038/s41598-017-07394-3)
Supplement: Supplementary file 1 — Supplementary info [file 41598_2017_7394_MOESM1_ESM.doc]

**SUPPLEMENTARY INFORMATION**

**TGFβ signaling is associated with changes in inflammatory gene expression and perineuronal net degradation around inhibitory neurons following various neurological insults**

Soo Young Kim, Vladimir V. Senatorov Jr., Christapher S. Morrissey, Kristina Lippmann, Oscar Vazquez, Dan Z. Milkovsky, Feng Gu, Isabel Parada, David A. Prince, Albert J. Becker, Uwe Heinemann, Alon Friedman, Daniela Kaufer

**SUPPLEMENTARY INFORMATION**

**Primer sequences.** The reference ribosomal subunit RNA, RPLP was used as an internal control. Primer sequences are as follows: Rplp, 5’- CCA AAG GTT TGG GAG AAC AA - 3’ and 5’- GGG TCA TGG CAT AGA GCA AT-3’; Serpine1, 5’- TCC TTT GGG ACA AAA CTG GAC GTG T -3’ and 5’- TGA GGT CTG GGA GGC TGG TTG G -3’; Stat3, 5’- ACC TCT TGA GTC CGT GAT GG -3’ and 5’- GGC GGA CAG AAC ATA GGT GT -3’; Timp1, 5’- GGA GTT TCT CAT CGC GGG CCG -3’ and 5’- CAC ACC CCA CAG CCA GCA CT -3’; Tenascin C, 5’- TGG TGC TGA ACG AAC TGC CC -3’ and 5’-TGG TTT TGG TAC GGA TGC TGG G -3’; Neurocan, 5’- AAG GAG CCA GCT CCA GTA TGG G -3’ and 5’- TGG TGT CCT GTG TGT CCT GAT CCC -3’; Ctgf, 5’- CGG AGC GTG ATC CCT GCG AC -3’ and 5’- GCT GCG GTA CAC GGA CCC AC -3’; Serpina3n, 5’- TCT GCA AAA CTG GAC CCT CTG-3’ and 5’- AGA GAC CCA CAG ACA GGC TC -3’.

**Antibodies used for Western Blot Analysis.**

*Primary antibodies* Goat anti-TIMP1 antibody, 1:8000, R&D system #AF580; mouse anti-STAT3, 1:1000, Cell Signaling #9310; rabbit anti-MMP9, 1:1000, Millipore AB19016; rabbit

rabbit anti-MMP14, 1:750, Abcam #AB51074; mouse anti-Albumin, 1:1500, Abcam #AB3781; anti-β-actin, 1:2000, Cell Signaling #4970 (used as the internal control for MMP9 and STAT3); rabbit anti-GAPDH, 1:2000 (used as the internal control for MMP14, Albumin, and TIMP1), Cell Signaling #2118.

*Secondary antibodies* Anti-goat IgG, HRP-linked, 1:1000, R&D Systems #HAF109; Anti-mouse IgG, HRP-linked, 1:2000, Cell Signaling #7076; Anti-rabbit IgG, HRP-linked, 1:2000, Cell Signaling #4970.

**SUPPLEMENTARY FIGURES**

**
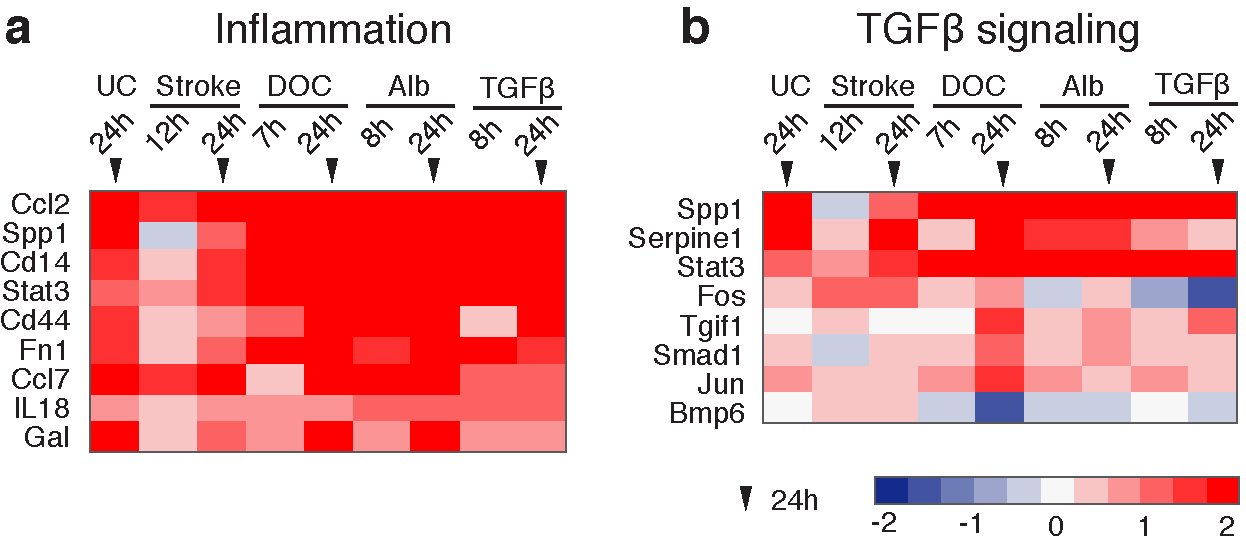
**

**Figure S1.** **Heat map displays of selected gene ontology terms.** Transcriptional profiles of undercut cortices (UC) and peri-infarct hippocampi (Stroke) were compared with those of rat cortices exposed to sodium deoxycholate (DOC, a BBB-disrupting agent), albumin (Alb), and TGFβ1(TGFβ) used in the previous study [^1^](#_ENREF_1). Comparisons were made with a total of nine conditions: peri-infarct hippocampi at 12 and 24 hours after cortical photothrombotic stroke operations, undercut cortices 24 hours after the partial cortical isolation, cortices 7 or 8 and 24 hours following BBB disruption induced by sodium deoxycholate (DOC), or direct cortical exposure to serum albumin or TGFβ1. The expression profiles were examined in selected gene ontology terms that were previously reported as enriched following BBB disruption, which include inflammation and TGFβ signaling. Genes related to inflammation, e.g., Ccl2, Ccl7, Cd14, and Gal, were found to be the most highly upregulated regardless of insult types, brain regions or time points examined. TGFβ-regulated inflammatory genes including Spp1, Serpine1, and Stat3 were consistently upregulated at 24 hour following all insults. Note that the common transcriptional activation is prominently found in inflammation. Heat map is indicated as a log_2_ scale.


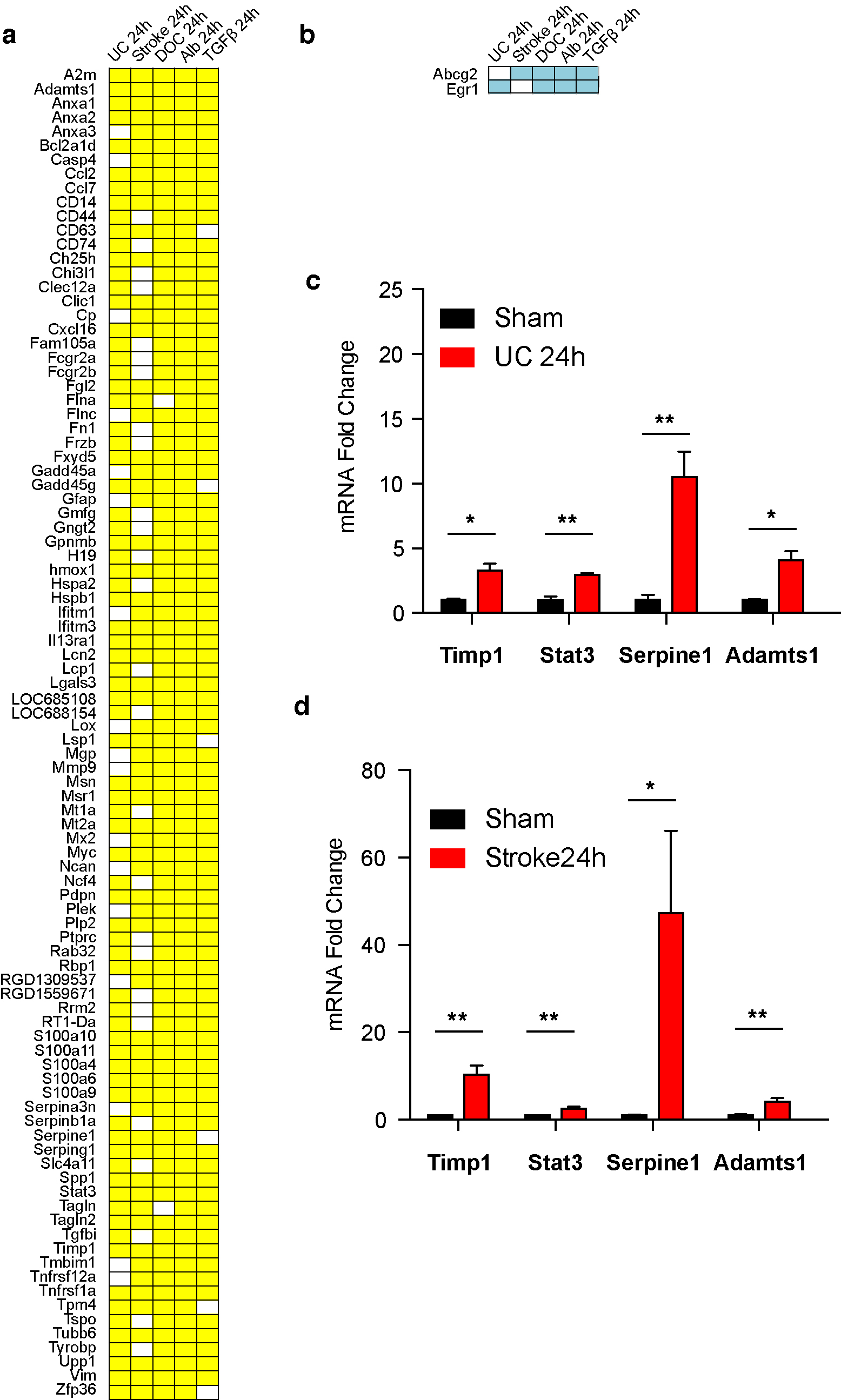


**Figure S2. Commonly regulated genes across five different models were selected.** (A, B) A yellow-colored rectangle indicate a vote scored for a differentially expressed gene (≥2-fold changes, A, upregulated; B, down-regulated) each condition. Quantitative real-time PCR analyses verified the expression levels of selected genes (C, D). mRNA levels of genes including Timp1, Stat3, Serpine1, and Adamts1 were examined in rat somatosensory cortex at 24h following the undercut operation (UC, n=3) compared to sham-operated animals (n=3; C) and in peri-infarct hippocampi 24 h after photothrombotic stroke (n=5) compared to sham-operated controls (n=5; D). Student t-tests (two-tailed) were used. *p < 0.05, **p < 0.01. Data are shown as mean ± S.E.

**
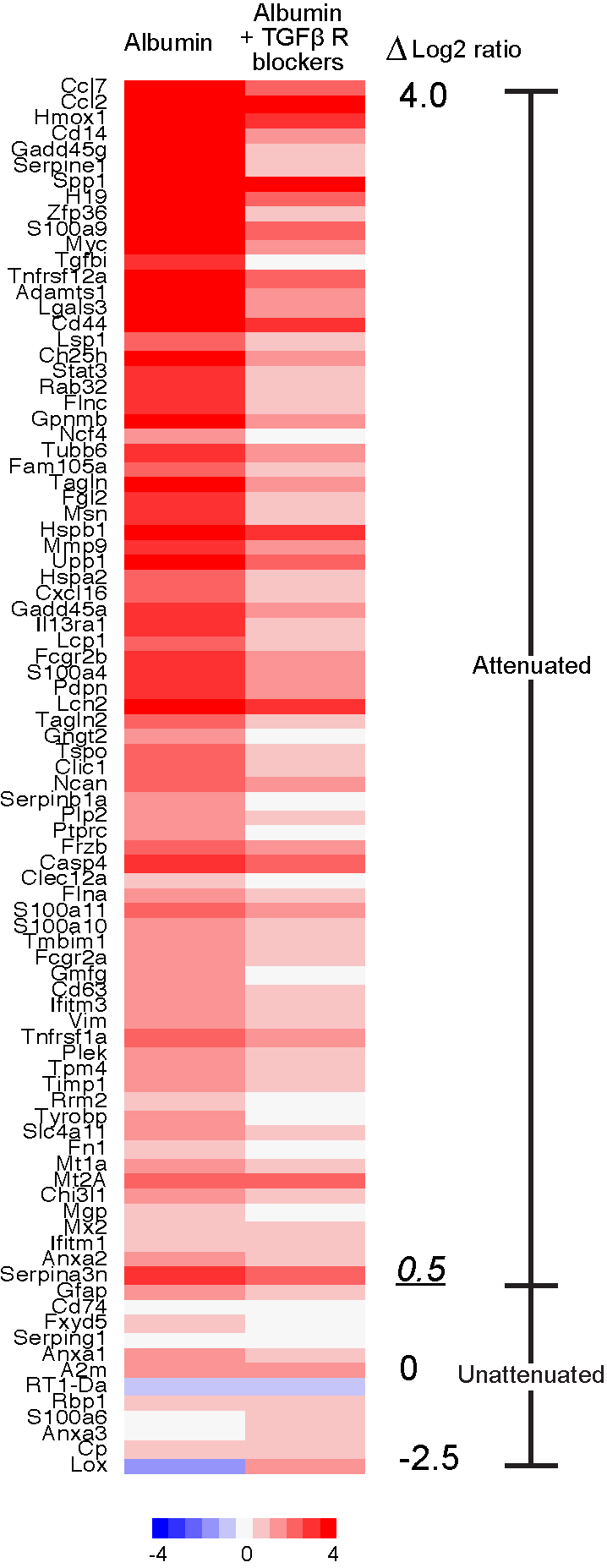
**

**Figure S3. The common transcriptional induction was highly dependent on TGFβ signaling.**

The expression levels of the commonly regulated 94 genes were examined in an additional microarray data set used in the previous study[^1^](#_ENREF_1) in that rat brains were collected 24 hours following either albumin exposure (n=3), or exposure to albumin plus a cocktail of TGFβ receptor blockers (a TGFβ type I receptor kinase inhibitor SB431542 and anti-TGFβ receptor II antibody; n=4). Of the 94 genes, 88 were found in this dataset and 85% of them were more than 2-fold upregulated following albumin exposure. The log_2_ ratio difference (∆) in the gene expression level upon treatment with albumin or albumin plus blockers was compared and a difference greater than 0.5 was considered to be attenuated by the blockers. Application of TGFβ blockers attenuated the effect of albumin in 94.7% of the upregulated genes. These results corroborate that the common transcriptional response is particularly dependent on TGFβ signaling. Heat maps are based on a log_2_ scale.

­
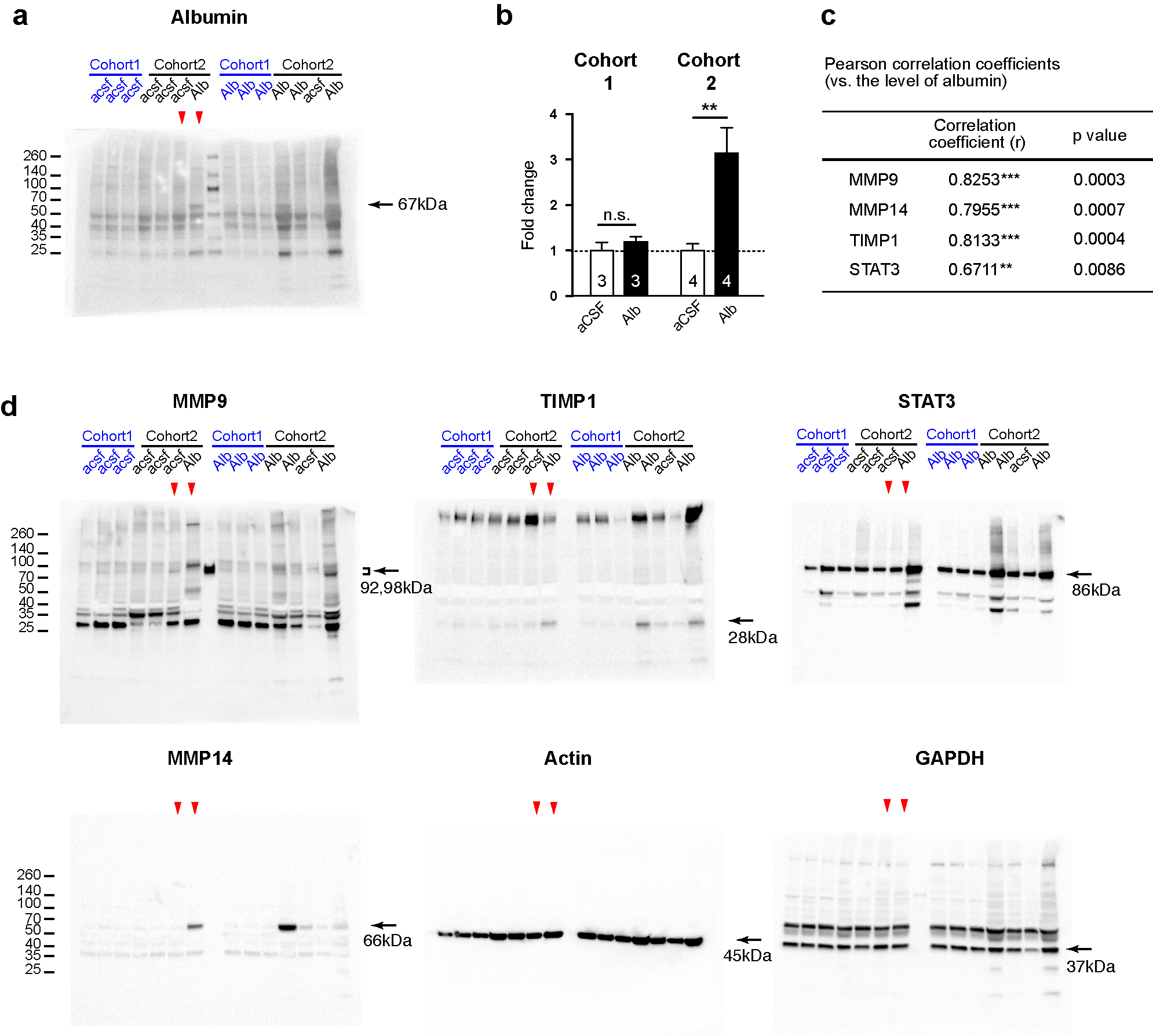


**Figure S4. Uncropped blots and the quantification of albumin in the hippocampus following osmotic pump implantation.** We performed two separate batches of surgeries implanting osmotic pumps for the sample preparation for western blot analysis (aCSF vs. Albumin, n=3 per group for Cohort 1, n=4 per group for Cohort 2). To verify the successful infusion of albumin, we quantified albumin expression in the dissected hippocampi acquired from two different cohorts by western blot analysis (A). While the amount of albumin found in hippocampi of animals infused with albumin was significantly increased compared to those infused with aCSF in Cohort 2, there was no group difference in the amount of albumin in Cohort 1 (B). Two way ANOVA was performed with Sidak’s post-hoc test (Interaction effect, F_(1,10)_ = 7.41, p < 0.05; Albumin (Alb) effect, F_(1,10)_ = 10.49, p < 0.01; Cohort effect F_(1,10)_ = 7.41, p < 0.05; Post-hoc Sidak’s test, aCSF vs. Alb: p = 0.93 in Cohort 1, p=0.002 in Cohort 2). (C) Pearson correlation coefficients for each protein with two-tailed p-value in a comparison with the amount of albumin are shown). MMP9, MMP14, TIMP1, and STAT3 proteins levels are strongly correlated with albumin amount in the individual brains of both cohorts. We interpret the lack of increased level of albumin in Cohort 1 as technical failure of the pump implantation, and henceforth continued with quantification of the effect of albumin in Cohort 2. Uncropped blots for both cohorts are presented here. Red arrowheads and black arrows indicate the columns and rows, respectively, of bands that are presented in Figure 5B.

**
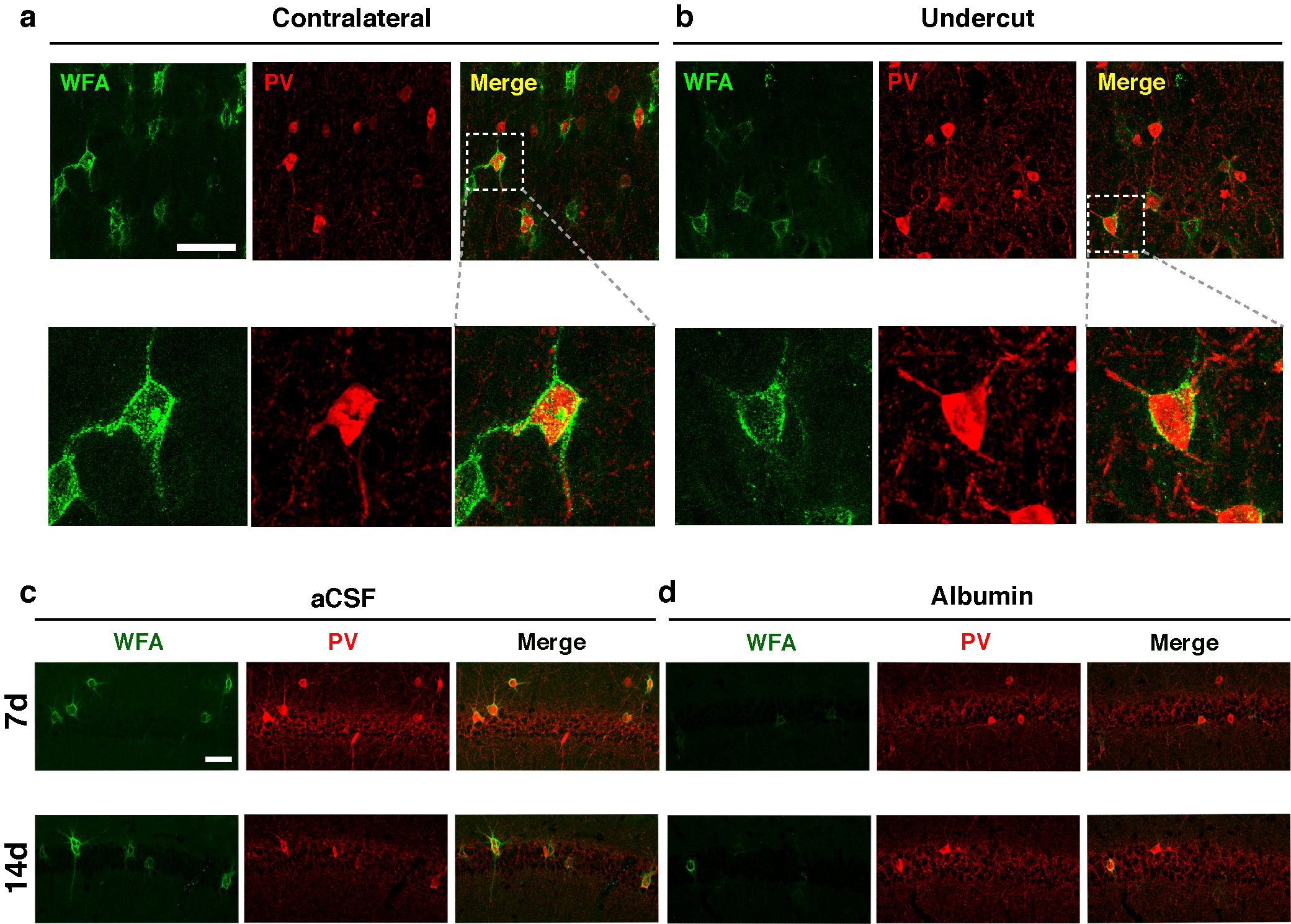
**

**Figure S5. PNNs around PV(+) interneurons following traumatic injury or albumin exposure.** (A-B) Representative confocal images of rat undercut or contralateral cortices stained for parvalbumin (PV) and perineuronal nets using *wisteria floribunda agglutinin* (WFA) 3 days after the operation. Areas within white rectangles in merged images (top) are shown magnified (bottom). (C-D) Representative confocal images of mouse hippocampal CA1 regions exposed to artificial cerebrospinal fluid (aCSF) or albumin via osmotic pumps for 7 days. Mice were sacrificed at 7 days (top) or 14 days post-implantation (bottom). Scale bar = 50 µm.

**SUPPLEMENTARY TABLES**

**Table S1. Summary of previous findings on pathological hyperexcitability** in rodent injury models

| Model | Photothrombotic cortical stroke | Partial cortical isolation (“Undercut”) | Albumin exposure | | BBB disruption by DOC | Cortical perfusion of TGFβ1 |
| --- | --- | --- | --- | --- | --- | --- |
|  |  |  | Cortical perfusion | ICV infusion |  |  |
| Region | Hippocampus | Partially isolated cortex | Cortex | Hippocampus | Cortex | Cortex |
| Excitation | No data^*^ | Enhanced connectivity[^2^](#_ENREF_2) | Increased[^3^](#_ENREF_3) | Synaptogenesis[^4^](#_ENREF_4) | Increased[^5^](#_ENREF_5) | No data |
| Inhibition | Decreased mRNA expression of GABA A receptor subunits [^6^](#_ENREF_6)  Reduced gamma oscillation[^6^](#_ENREF_6) | Reduced synaptic inhibition and reorganization of inhibitory synaptic circuits [^7^](#_ENREF_7)  Abnormal fast-spiking PV(+) interneurons[^8^](#_ENREF_8) | Decreased GABAergic transmission and mRNA expression of inhibitory synapse-related genes | No changes in the number of somatic and dendritic inhibitory synapse[^4^](#_ENREF_4) | Decreased mRNA expression of inhibitory synapse related genes[^1^](#_ENREF_1) | Decreased mRNA expression of inhibitory synapse related genes[^1^](#_ENREF_1) |
| Neuronal hypersynchrony | Spreading depolarization in peri-infarct hippocampus[^9^](#_ENREF_9)  Epileptiform discharge in peri-infarct hipppomcampus  Recurring seizures[^6^](#_ENREF_6) | Epileptiform activity beginning as early as 3 days[^10^](#_ENREF_10), prominent 7-14 days[^11^](#_ENREF_11) following undercut  Recurring seizures[^11^](#_ENREF_11) | Epileptiform activity  Recurring seizures[^14^](#_ENREF_14) | Abnormal homo- and heterosynaptic plasticity[^15^](#_ENREF_15)  Epileptiform activity  Recurring seizures[^4^](#_ENREF_4) | Epileptiform activity  Recurring seizures[^14^](#_ENREF_14) | Epileptiform activity[^1^](#_ENREF_1) |

^*^Excitatory synaptogenesis has been repeatedly demonstrated in the peri-infarct region following stroke .

ICV, Intracerebroventricular; DOC, Sodium deoxycholate; PV, Parvalbumin.

**Table S**2 ­­ Clinical data of human subjects

| **Autopsy age-matched Controls** | | | | |
| --- | --- | --- | --- | --- |
| Gender | Age at death (yrs) | Cause of Death | Neuropathological diagnosis | Staining |
| F | 36 | Acute failure of liver function of unclear cause | No evident neuropathological alterations | GFAP/pSmad |
| M | 26 | T-cell lymphoma with ARDS |  | GFAP/pSmad  PV/WFA |
| M | 33 | M. Crohn with multiple gut necrosis |  | GFAP/pSmad  PV/WFA |
| F | 63 | Perforated colon carcinoma, peritonitis and cardiac arrest |  | PV/WFA |
| F | 61 | Coronary artery sclerosis based cardiac arrest |  | PV/WFA |
| **Temporal lobe epilepsy patients** | | | | |
| Gender | Age at surgery (yrs) | Clinical diagnosis | Neuropathological diagnosis | Staining |
| F | 61 | Bilateral pharmacoresistant convulsive seizures since several years, on MRI hippocampal sclerosis on the left side | Hippocampal sclerosis | PV/WFA |
| M | 48 | Focal pharmacoresistant seizures and cognitive impairment since several years, on MRI hippocampal sclerosis on the left side |  | PV/WFA |
| M | 31 | Focal seizures with origin in the right temporal lobe |  | GFAP/pSmad  PV/WFA |
| M | 18 | Focal, occasionally bilateral pharmacoresisatant convulsive seizures since several years, on MRI hippocampal sclerosis on the left side |  | GFAP/pSmad  PV/WFA |
| F | 23 | Focal and generalized seizures with origin in the right temporal lobe |  | GFAP/pSmad |
| F | 34 | Focal seizures with origin in the left temporal lobe and HS on MRI |  | GFAP/pSmad |
| M | 22 | Pharmacoresistant epilepsy, on MRI hippocampal sclerosis on the right side |  | GFAP/pSmad |

ARDS, acute respiratory distress syndrome; MRI, magnetic resonance imaging.

**REFERENCES**

1. Cacheaux LP*, et al.* Transcriptome Profiling Reveals TGF-beta Signaling Involvement in Epileptogenesis. *Journal of Neuroscience* **29**, 8927-8935 (2009).

2. Jin X, Prince DA, Huguenard JR. Enhanced excitatory synaptic connectivity in layer v pyramidal neurons of chronically injured epileptogenic neocortex in rats. *J Neurosci* **26**, 4891-4900 (2006).

3. David Y*, et al.* Astrocytic Dysfunction in Epileptogenesis: Consequence of Altered Potassium and Glutamate Homeostasis? *Journal of Neuroscience* **29**, 10588-10599 (2009).

4. Weissberg I*, et al.* Albumin induces excitatory synaptogenesis through astrocytic TGF-beta/ALK5 signaling in a model of acquired epilepsy following blood-brain barrier dysfunction. *Neurobiol Dis* **78**, 115-125 (2015).

5. Tomkins O*, et al.* Blood-brain barrier disruption results in delayed functional and structural alterations in the rat neocortex. *Neurobiol Dis* **25**, 367-377 (2007).

6. Lippmann K*, et al.* Epileptiform activity and spreading depolarization in the blood–brain barrier-disrupted peri-infarct hippocampus are associated with impaired GABAergic inhibition and synaptic plasticity. *Journal of Cerebral Blood Flow & Metabolism*, (2016).

7. Jin X, Huguenard JR, Prince DA. Reorganization of inhibitory synaptic circuits in rodent chronically injured epileptogenic neocortex. *Cereb Cortex* **21**, 1094-1104 (2011).

8. Ma Y, Prince DA. Functional alterations in GABAergic fast-spiking interneurons in chronically injured epileptogenic neocortex. *Neurobiol Dis* **47**, 102-113 (2012).

9. Lapilover EG*, et al.* Peri-infarct blood-brain barrier dysfunction facilitates induction of spreading depolarization associated with epileptiform discharges. *Neurobiol Dis* **48**, 495-506 (2012).

10. Takahashi DK, Gu F, Parada I, Vyas S, Prince DA. Aberrant excitatory rewiring of layer V pyramidal neurons early after neocortical trauma. *Neurobiol Dis* **91**, 166-181 (2016).

11. Graber KD, Prince DA. Chronic partial cortical isolation. In: *Models of Seizures and Epilepsy* (ed^(eds Pitkanen A, Schwartzkroin P, Moshe S). Elsevier Academic Press (2006).

12. Ivens S*, et al.* TGF-beta receptor-mediated albumin uptake into astrocytes is involved in neocortical epileptogenesis. *Brain* **130**, 535-547 (2007).

13. Seiffert E*, et al.* Lasting blood-brain barrier disruption induces epileptic focus in the rat somatosensory cortex. *J Neurosci* **24**, 7829-7836 (2004).

14. Bar-Klein G*, et al.* Losartan prevents acquired epilepsy via TGF-beta signaling suppression. *Ann Neurol* **75**, 864-875 (2014).

15. Salar S*, et al.* Synaptic plasticity in area CA1 of rat hippocampal slices following intraventricular application of albumin. *Neurobiol Dis* **91**, 155-165 (2016).

16. Murphy TH, Corbett D. Plasticity during stroke recovery: from synapse to behaviour. *Nature Reviews Neuroscience* **10**, 861-872 (2009).

17. Kim SY*, et al.* Experience with the "good" limb induces aberrant synaptic plasticity in the perilesion cortex after stroke. *J Neurosci* **35**, 8604-8610 (2015).
